# From bedside to bench: New insights in epilepsy‐associated tumors based on recent classification updates and animal models on brain tumor networks

**DOI:** 10.1002/1878-0261.13680

**Published:** 2024-06-20

**Authors:** Silvia Cases‐Cunillera, Lea L. Friker, Philipp Müller, Albert J. Becker, Gerrit H. Gielen

**Affiliations:** ^1^ INSERM U1266, Neuronal Signaling in Epilepsy and Glioma Institute of Psychiatry and Neuroscience of Paris (IPNP), Université Paris Cité Paris France; ^2^ Section for Translational Epilepsy Research Institute of Neuropathology, University Hospital Bonn Bonn Germany; ^3^ Institute of Neuropathology University Hospital Bonn Bonn Germany

**Keywords:** brain tumors, epilepsy, *in utero* electroporation, mouse model, neuronal activity

## Abstract

Low‐grade neuroepithelial tumors (LGNTs), particularly those with glioneuronal histology, are highly associated with pharmacoresistant epilepsy. Increasing research focused on these neoplastic lesions did not translate into drug discovery; and anticonvulsant or antitumor therapies are not available yet. During the last years, animal modeling has improved, thereby leading to the possibility of generating brain tumors in mice mimicking crucial genetic, molecular and immunohistological features. Among them, intraventricular *in utero* electroporation (IUE) has been proven to be a valuable tool for the generation of animal models for LGNTs allowing endogenous tumor growth within the mouse brain parenchyma. Epileptogenicity is mostly determined by the slow‐growing patterns of these tumors, thus mirroring intrinsic interactions between tumor cells and surrounding neurons is crucial to investigate the mechanisms underlying convulsive activity. In this review, we provide an updated classification of the human LGNT and summarize the most recent data from human and animal models, with a focus on the crosstalk between brain tumors and neuronal function.

## Graphical abstract

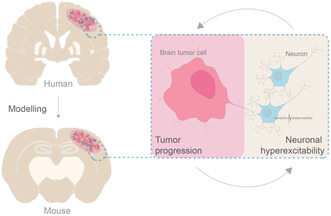

**Abbreviations**
DLGNT: diffuse leptomeningeal glioneuronal tumor
DNT: dysembryoplastic neuroepithelial tumor
GG: ganglioglioma
GNT: glioneuronal tumor
HGG: high‐grade glioma
IUE: *in utero* electroporation
LGNT: low‐grade neuroepithelial tumor
MVNT: multinodular and vacuolating neuronal tumor
PDX: patient‐derived xenograft
RGNT: rosette‐forming glioneuronal tumor

## 1 Introduction

Low‐grade neuroepithelial tumors (LGNTs), also known as long‐term epilepsy‐associated tumors (LEATs), are generally benign and tend to occur in children. Among them, glioneuronal tumors (GNTs), in particular gangliogliomas (GGs) and dysembryoplastic neuroepithelial tumors (DNTs), represent the majority of cases [1, 2]. However, the classification and diagnosis of these tumors can be challenging due to a broad overlap of specific molecular signatures. Here, we will summarize the 2021 Central Nervous System (CNS) World Health Organization (WHO) classification of epilepsy‐associated neuroepithelial tumors [3] and discuss the state‐of‐the‐art of neuropathological and molecular diagnostics, highlighting that common MAPK pathway alterations are found overlapping in different LGNT types. Therefore, the combination of cytological, histomorphological, genomic, genetic and epigenetic features is essential for modern precision diagnostics [4].

The current lack of tailor‐made antitumor and antiseizure medication for LGNT urges the need for studying mechanisms underlying neuronal hyperexcitability related to the neoplastic lesion. Until now, most of the studies relied on high‐grade glioma (HGG) animal models based on intracranial patient‐derived xenografts (PDX) to study the interplay between tumor cells and neuronal network function. The use of these PDX‐derived animal models provided evidence that the tumor cells can trigger alterations of the neuronal network including neuronal hyperexcitability. In turn, a prominent role of neuronal activity in tumor growth has become increasingly evident. Particularly, a collection of data provided by the research group of Michelle Monje clearly shows that neuronal activity can enhance glioma progression in a paracrine as well as synaptic fashion [5, 6, 7]. Although these data contribute to very notorious and fundamental knowledge for understanding the neuronal networks in the field of neuro‐oncology, reducing seizures does not represent the main focus in the field of HGGs. Instead, the main therapeutic goal is to reduce tumor progression and improve prognosis.

A different pathological scenario is expected for LGNTs, in which epilepsy is the main and often the only symptom. In this regard, PDXs represent many limitations for the investigation of neuron‐tumor interactions in LGNTs. Among them, the interaction between the transplanted tumor cells (human) and the preexisting neurons (mouse) does not intrinsically occur along tumor progression, which prevents the study of long‐term epilepsy associated with these slow‐growing brain tumors. Indeed, modeling the intrinsic connections between the tumor and the tumor microenvironment (TME) of LGNT models is critical to study the underlying seizure activity. Such processes can be accurately mirrored in developmental brain tumor models by using intraventricular *in utero* electroporation (IUE), which is a technique that allows genetic manipulation of neural precursor cells *in vivo* [8].

Molecular mechanisms sustaining interaction between tumor and surrounding neurons in LGNTs are starting to be elucidated. In this review, we will summarize the most striking tumor‐related mechanisms that could contribute to seizures and we highlight the importance of the TME in the generation of convulsive activity.

Taken together, the proper classification of LGNTs is essential for the generation of suitable animal models and for the stratification of their features. Data from PDX‐based animal models of HGG are useful to understand the basic interaction between neoplastic and neuronal cells. For the study of LGNTs, IUE appears as a proper tool to generate brain tumors recapitulating many features of their human counterparts. Studies on IUE‐based animal models are required to understand the crosstalk between brain tumor cells and preexisting neuronal structures. Revealing how these interactions occur may be the basis for finding new molecules that are targetable to ultimately reduce seizure activity and suppress tumorigenesis.

## 2 Epilepsy‐associated (low‐grade) neuroepithelial tumors

### 2.1 The 2021 CNS WHO classification and state‐of‐the‐art in neuropathological/molecular diagnostics

**Fig. 1:**
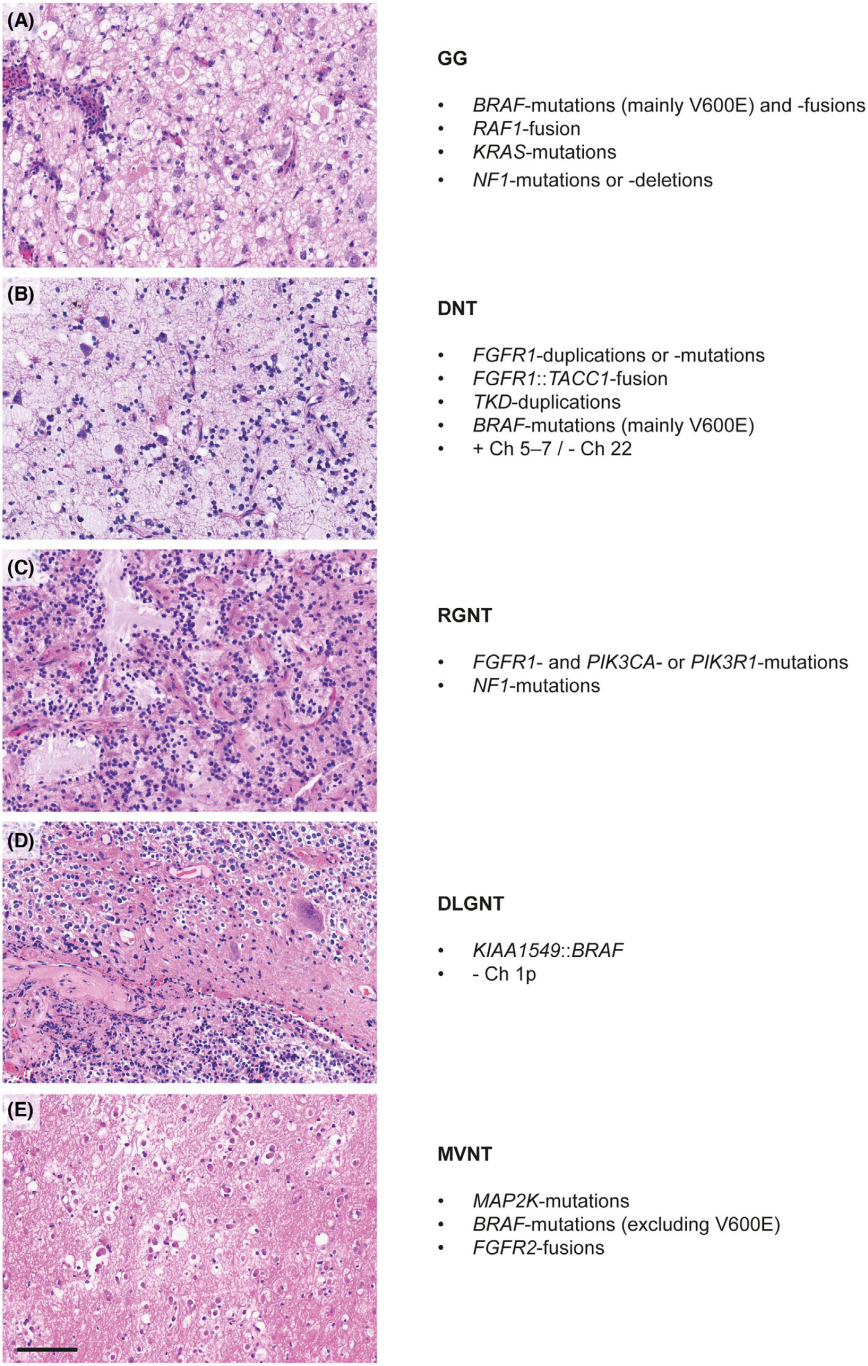
Histomolecular characteristics of common LGNTs associated with lesional epilepsies. (A) GG, (B) DNT, (C) RGNT, (D) DLGNT and (E) MVNT. On left, the H&E‐stained slides are displayed, showing the typical histological appearance of each individual tumor type. To the right molecular genetic hallmarks are listed completing the diagnostics. Scale bar equates 200 μm. GG, ganglioglioma; DLGNT, diffuse leptomeningeal glioneuronal tumor; DNT, dysembroplastic neuroepithelial tumor; MVNT, multinodular and vacuolating neuronal tumor.

Starting with the 2016 update of the WHO CNS Tumor Classification and further extended in the 2021 WHO CNS Tumor Classification, molecular profiling has become central in the diagnostics of brain tumors [9]. The current morpho‐molecular approach in daily routine in neuropathological diagnostics has substantially enhanced our comprehension of primary pathogenic mechanisms of brain tumors and steadily led to the definition of new tumor types, for which multiomic integration is shown to increase the diagnostic accuracy of multiple CNS tumors [4]. Over the last decade, genetic and epigenetic findings have also changed the understanding of low‐grade neoplasms involved in epilepsy. Histomorphologically, especially LGNTs can appear very similar. Also, molecular characteristics show a certain overlap between different low‐grade glioneuronal tumor types. The most frequently mutant gene in lesional epilepsies is *BRAF* [10]. *BRAF* alterations are commonly observed in GGs, DNTs, diffuse leptomeningeal glioneuronal tumors (DLGNTs) and multinodular and vacuolating neuronal tumors (MVNTs). However, the interpretation of histopathological, immunohistochemical, and molecular genetic findings in synopsis usually allows to assign a clear diagnosis, but can be challenging. In the following paragraphs, the histomolecular characteristics of the most common epilepsy‐associated LGNTs are described [3]. A summary is provided in Fig. 1 and Table 1.

**Table 1:** Selected low‐grade GNTs defined in the 2021 WHO classification of the tumors of the central nervous system.

Tumor type	CNS WHO grade
Ganglioglioma (GG)/Gangliocytoma	1
Desmoplastic infantile ganglioglioma/astrocytoma (DIG/DIA)	1
Dysembroplastic neuroepithelial tumor (DNT)	1
*Diffuse glioneuronal tumor with oligodendroglioma‐like features and nuclear clusters*	Provisional tumor type; no grading assigned
Papillary glioneuronal tumor (PGNT)	1
Rosette‐forming glioneuronal tumor (RGNT)	1
Myxoid glioneuronal tumor	1
Diffuse leptomeningeal glioneuronal tumor (DLGNT)	2 or 3
Multinodular and vacuolating neuronal tumor (MVNT)	1

GG (CNS WHO grade 1) (Fig. 1A) is defined as a slowly growing glioneuronal tumor composed of dysmorfic ganglion and neoplastic glial cells. Molecularly, it is characterized by genetic alterations that lead to the activation of the MAPK pathway as *BRAF* mutations (mainly V600E) or fusions, *RAF1* fusion, *KRAS* mutation and *NF1* mutation or deletion.

*FGFR1* alterations are considered to be the main molecular driver of DNT (Fig. 1B) pathology. *FGFR* duplications or mutations as well as *FGFR1::TACC1* fusions are characteristic of DNTs, although they are not specific. In comparison, *TKD* duplications are known to be relatively specific to DNT. Furthermore, *BRAF* mutations and copy number aberrations with a gain of chromosomes 5–7 and a loss of chromosome 22 have been reported. Histologically, DNTs typically show a multinodular pattern. Cells of the pathognomonic glioneuronal element are often grouped in columns, aligned perpendicularly to the surface. Another typical histological hallmark of DNTs is the presence of so‐called ‘floating neurons’ in a mucinous matrix.

Rosette‐forming glioneuronal tumors (RGNTs) (Fig. 1C) show a biphasic neurocytic and glial architecture of lower to moderate cellularity. Neurocytic rosettes and/or perivascular pseudorosettes are commonly observed. The glial component typically dominates the overall appearance and may show microcystic matrix loosening. Typical molecular alterations in RGNT are *FGFR1* mutation in combination with either *PIK3CA* or *PIK3R1* mutation. In addition, *NF1* mutation can occur.

As the name implies, DLGNTs (Fig. 1D) grow inside the leptomeninges. They mostly affect the spinal cord and less frequently appear in the cerebral hemispheres. When infiltrating CNS parts, the intraparenchymal component may resemble DNTs. DLGNTs are composed of mostly monomorphic oligodendrocyte‐like tumor cells. The tumor matrix may show desmoplastic and myxoid changes. Among *KIAA1549::BRAF* fusions, molecular analysis of DLGNT reveals a loss of chromosome arm 1p. Contrarily, in the case of missing 1p deletion, the diagnosis of DLGNT is unlikely.

Tumor cells of MVNTs (Fig. 1E) characteristically show vacuolization of their cytoplasm or pericellular space. They are usually low in count and grow in nodular patterns. Observed genetic changes commonly activate the MAPK pathway, most typically by alterations in *MAP2K1* gene, followed by *BRAF* mutations and fusions implicating *FGFR2*.

Especially in cases with diagnostic uncertainty, an initial molecular workup is often essential to avoid misdiagnoses. Therefore, the technical equipment for DNA methylation profiling, targeted gene panel sequencing and gene fusion analysis have to be considered crucial in modern neuropathological routine diagnostics.

### 2.2 From LEATs to new concepts: Current and future directions

Generally, the terminological system of brain tumors has been based on ontological aspects. This held particularly true for gliomas, being the most prevalent group of adult brain neoplasms as it was perceived that they develop from differentiated non‐neuronal neuroepithelial cells, especially astrocytes and oligodendroglia. Thus, in nosological regards, the LEAT system was disruptive, since it emphasized a clinical aspect as a key denominator of a group of brain neoplasms. The LEAT system, introduced by Johannes Schramm and collaborators, was based on the observation that generally rare tumor entities are accumulated in patients with pharmacorefractory long‐term epilepsy that do not match with the WHO nomenclature and grading system but encounter neurodevelopmental entities with emphasis on the GG and DNT spectrum [11]. Shared LEAT features are given by seizure onset at adolescence, frequent temporal lobe localization, generally benign biological behavior, virtual absence of common genetic findings in diffuse gliomas (*IDH1* ^*R132H*^, 1p/19q co‐deletions) but parallel frequent alterations in *BRAF*, *FGFR1*, *FGFR2*, *MYB/L1* and *PRKCA*. Additionally, in practical regards, there is a problematic low inter‐ and intra‐rater agreement on these entities [12].

Considering the frequent association with recurrent spontaneous seizures, the understanding of key epileptogenic pathomechanisms represents a particular challenge of LEATs. Therein, the ‘tumorocentric’ concept emphasizes the epileptogenic role of neurons, as it has been demonstrated for neurons electroporated with a mouse *BRAF* ^*V600E*^ analog *in vivo* [13]. The ‘epileptocentric’ concept instead emphasizes that the peritumoral neocortical microenvironment is key for tumor‐related epileptic activity, due to neoplasm‐related impaired Glutamate‐ and GABA‐ergic dynamics contributing to epileptogenicity [14, 15].

The 2021 WHO CNS Tumor Classification has been revolutionary with respect to the relevance of molecular examination results in the classification of frequent brain neoplasms still including traditional cyto−/histological and immunohistochemical assays. Almost 30% of the newly introduced entities comprise low‐grade glioneuronal or glial entities. Thus, it emphasizes the importance of integrated, combinatorial paths to diagnosis and layered reports with a strong role in novel diagnostic technologies such as DNA methylome profiling [3]. For the LEAT spectrum, there is a substantial need to extent the use of molecular genetic diagnostic tools in concert with a histomorphology‐based classification to specify clinically meaningful tumor entities. However, the 2021 WHO CNS Tumor Classification carefully defines molecular‐pathologic features whereas the epileptological sequelae are only somewhat sparsely considered. It is an ongoing task to introduce the nosological system for clinical epileptologists. Histological déjà‐vus, furthermore, suggest that recent WHO classification system introduced ‘novel’ entities reflect pre‐described neoplasms out of the LEAT spectrum; in an excellent overview on this intricate topic [16] Ingmar Blümcke outlines the prominent example of the diffuse astrocytoma, MYB‐ or MYBL1‐altered, which presumably has been firstly described as ‘isomorphic astrocytoma’ 20 years ago by him and colleagues [17]. The lack of sufficiently large and multicenter, prospectively collected patient cohorts with LEATs underline the importance of mouse LEAT models.

## 3 Animal models on the interaction between low‐grade GNTs and adjacent neurons

### 3.1 Tumor xenograft mouse models for tumor‐neuron interaction: New insights from HGGs

#### 3.1.1 PDX‐based models of malignant glioma

Interactions between tumor cells and neurons have been extensively studied in animal models for HGGs based on intracranial PDXs, in which immortalized human‐derived tumor cells are transplanted in the brain of immunocompromised mice [18, 19, 20, 21]. Due to the bad prognosis of HGGs (classified as grade 3 and 4 by the WHO; [3]), most of the investigations in this field are focused on reducing tumor progression and increasing survival rate. In this context, the rapid growth of the xenografts engrafted within the mouse brain reproduces the fast progression, malignancy, and invasion of HGGs. Another major advantage is the possibility to label the transplanted cells and thereby monitor their dynamics, infiltration and interactions within the mouse brain tissue [22, 23]. Indeed, this provides a convenient tool to evaluate and track the *de novo* connections between engrafted tumor cells and murine preexisting brain structures, which serve as TME.

#### 3.1.2 From tumor to neurons

In this line, the crosstalk between tumors and neurons is important in the field of epilepsy in neuro‐oncologic lesions. Epileptic seizures have been identified in PDX mouse models of HGGs [24, 25] which provided new insights into the tumor‐related mechanisms related to seizure activity. An imbalance between excitation and inhibition in the tumor as well as TME arises as a key mechanism involved in this association. It is well known that tumor cells can change the expression of glutamate transporters leading to defective extracellular glutamate homeostasis. As such, glioma cells upregulate the expression levels of the antiporter system xCT which mediates glutamate release to the extracellular space in exchange for cystine uptake [26, 27, 28, 29, 30]. Upregulation of this glutamate exchanger system xCT has been associated with seizures in both humans [31, 32, 33] as well as PDX mouse models of glioma [25, 34].

In addition to a higher release of glutamate, a decrease in its reuptake has also been observed. The excitatory amino acid transporters (EAAT) 1 (GLAST) and EAAT2 (GLT‐1) are responsible for the majority of the glutamate uptake in the brain [35, 36, 37]. These transporters show reduced expression in glioma cells [26, 33, 38, 39, 40] and glioma human tissue samples [39, 41, 42].

Accumulating evidence also implicates GABA‐ergic disinhibition in the pathology of tumor‐associated epilepsy. The inhibitory or excitatory function of the GABA neurotransmitter depends on the chloride equilibrium potential being defined by the expression ratio of the chloride intruder NKCC1 and the chloride extruder KCC2 (reviewed in [43, 44]). Of importance, a decrease in the function of the KCC2 transporter has been reported in PDX mouse models of glioma [45] as well as in human gliomas [46, 47, 48] and alterations in the expression ratio of these transporters have profound effects on seizure activity [49].

#### 3.1.3 From neurons to tumors

Besides the tumor‐derived effects mentioned above, a prominent role of neuronal activity in tumor growth has become increasingly evident. Venkatesh and colleagues were the first to show that neuronal activity harbors the potential to promote glioma progression through the activity‐mediated release of neuroligin‐3 (NGLN3) [5]. A follow‐up study revealed that glioma growth is prevented in *NGLN3* ^*KO*^ mice, which further supported the implication of NGLN3 as a crucial mediator [6]. In addition, there is also evidence showing that neurons communicate synaptically with glioma cells stimulating tumor growth and progression [7, 50].

Collectively, these data point towards a new concept by which glioma cells and neurons can communicate in a bidirectional manner: tumor cells can perturb neuronal network activity and, in turn, neurons can influence glioma progression. Nevertheless, the main therapeutic goal of HGGs is not focused on reducing seizures (50% of patients with HGG suffer from epileptic seizures; [51]) but instead, on decreasing/slowing the tumor progression and ameliorating its prognosis (median survival time 13–36 months depending on grading; [52, 53, 54]).

In contrast, epilepsy represents the main, and often the only, symptom in patients with LGNTs. These neoplasms are generally benign and tend to occur in children. Among them, GNTs, in particular, GGs and DNTs, represent the majority of cases [1, 2, 55, 56, 57, 58, 59].

The reasons behind this strong association with epilepsy are suggested to be multifactorial. In general, slow‐growing neoplasms harbor higher epileptogenic features than HGGs [56, 58, 59, 60, 61, 62]. Moreover, the presence of a neuronal tumor component within the GNT with a preserved expression of neuroactive molecules [63, 64, 65, 66, 67, 68] may contribute to the higher propensity to epileptogenic potential compared to other types of gliomas. Additionally, the high association of LGNTs with adjacent dysplastic cortical structures [56, 64, 69, 70, 71] suggests that these lesions may arise from the same alteration occurring during brain development (reviewed in [72, 73]). Indeed, the high incidence of LGNTs in children supports that they may originate during embryonic time periods. Considering that brain development is a strictly regulated period that is crucial for the formation of correct neuronal circuits [74, 75, 76, 77], it is thus not surprising that alterations during this time window may be more prone to trigger ictogenesis.

The spectrum of involved genetic markers may also help to explain this high association with seizure activity. Contrary to HGGs, which mainly rely on mutations in the histone H3.3 (particularly in pediatric HGGs, [78, 79]), *IDH1/2*, *PDGFRA*, *EGFR*, *PTEN* or *CDKN2A/B* [3, 80, 81, 82, 83], LGNTs are defined by a different group of genetic mutations including MAPK signaling pathway activation (e.g., *BRAF*, *FGFR*), *MYB* or *PRKCA* [84, 85, 86, 87]. For instance, *BRAF* ^*V600E*^, which is frequently found in GNTs, has been associated with worse recurrence‐free survival [88, 89] and postoperative seizure outcome [90, 91]. Also, the PI3K‐mTOR signaling pathway is frequently over‐activated in human GGs [91, 92] and has been shown to contribute to epileptogenesis in these tumors [90].

Overall, these multiple lines of evidence highlight the urgent need for finding anticonvulsant therapies for LGNTs, in particular for GNTs, and thus it seems worthwhile to study mechanisms behind the dialog between tumor cells and neurons as it has been performed in HGG models.

### 3.2 Application to LGNTs: Intrinsic connections with the tumor microenvironment

#### 3.2.1 PDX approach for modeling LGNT

As previously mentioned, PDX models are reasonably accurate for HGGs, which allows the preservation of the genetic and histological complexity of primary gliomas. However, the generation of PDX models for LGNTs is hindered by the very low rate of engraftment in the mouse brain [93]. This is evidenced by the very few studies that could model LGNT *in vivo* relying on the PDX model [94, 95, 96]. Additionally, the limitations in accessibility and the lack of enough tissue specimens (due to the low number of cases) make the PDX model for LGNTs difficult to use for large‐scale studies.

Beyond the technical challenge, PDXs represent many limitations for the investigation of neuron‐tumor interactions in slow‐growing tumors. First, the interaction between the transplanted tumor cells (human) and the preexisting neurons (mouse) does not intrinsically occur along tumor progression, which hampers the study of long‐term epilepsy associated with slow‐growing brain tumors. Contrarily to malignant gliomas in which seizures may be elicited due to direct mechanical tumor mass effects, pressure, ischemia, edema or blood–brain barrier disruption [97, 98, 99], the slow proliferating rate of LGNTs [100, 101, 102] intuitively suggests that epilepsy may be triggered by the slow and progressive integration of the neoplastic structures into the neuronal networks. Additionally, a fundamental disadvantage of the PDX models is the lack of a preserved immune system as the engraftment needs to be performed in immunodeficient mice to avoid an immune reaction against the transplanted tumor tissue [103, 104]. This aspect presents a formidable challenge for the successful study of the natural immune response, particularly, taking into account that the immune system may contribute to neuronal hyperexcitability in LGNTs [105, 106, 107, 108]. Finally, the species‐derived differences between the mouse and human brain highlight the need to use other animal models for LGNT with naturally occurring cancers.

Given the high propensity to seizure activity in LGNTs, finding new anticonvulsant therapies is the main objective [57, 101]. Even if the comprehension of epilepsy‐associated tumors is continuously improving, no anticonvulsant or antitumor therapies are available yet. Until recently, the limited availability of animal models that developed fully penetrant LGNTs has restricted progress in its research.

#### 3.2.2 IUE‐based models of LGNTs

**Fig. 2:**
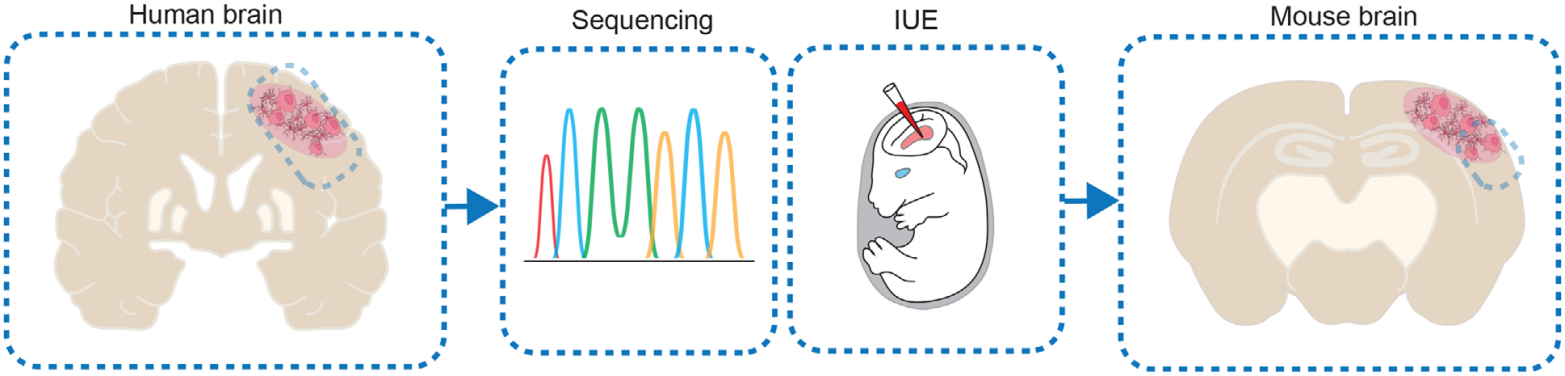
Modeling developmental brain tumors in mice. Based on molecular and genetic findings from human brain tissue, developmental brain tumors can be modeled in mice using IUE methodology. IUE, *in utero* electroporation.

Intraventricular IUE is a technique that allows genetic manipulation of neural precursor cells *in vivo* [8, 109, 110, 111, 112] and serves as a valuable tool for the generation of animal models for neurodevelopmental studies [113, 114, 115, 116, 117, 118, 119]. Several aspects support that the IUE technique has advantages over intracranial PDX models for the generation of animal models for epilepsy‐associated LGNTs. A schematic overview is provided in Fig. 2. First, (1) tumor cells in the brain evolve *in situ* in their endogenous TME, allowing the natural course of tumor initiation and progression. This is highly relevant for modeling slow‐growing brain tumors with long‐lasting interactions with preexisting neuronal structures. Moreover, (2) a key advantage of the IUE tumor models is that the immune TME remains intact, which enables the study of immune‐related responses accompanying these neoplastic lesions [91, 120, 121, 122]. Additionally, (3) the IUE technique offers an approach to model tumors that are suggested to originate during brain development. Particularly, this strategy is suited to (4) mirror histopathological traits of GNTs due to the possibility to genetically alter neural cell precursor populations still capable of differentiating to both neuron and glial cell lineages as it has been suggested to occur in human GGs [101, 123]. In this context, the combination of IUE with the ‘piggyBac’ (PB) transposon system offers the possibility to integrate transgenes into the genome of the cells located at the subventricular zone, thereby targeting the whole lineage of neural progenitors *in vivo* [124].

Overall, the aforementioned aspects highlight the advantage of PB‐IUE as a technique to use for the generation of animal models for developmental neoplasms, in particular for GNTs. To date, several IUE‐based animal models for brain tumors exist for both LGNTs and HGGs (Table 2). However, despite the substantial progress achieved in the generation of IUE‐dependent animal models, only two studies have generated and characterized tumor animal models for LGNT, more specifically for GGs [13, 125].

**Table 2:** List of the existing animal models for HGG and LGNT based on PB‐IUE. Animal modeling based on data from studies on humans aims to recapitulate primary developmental brain tumor features and serve as a tool to study the pathogenesis of epilepsy‐associated tumor processes, particularly tumor progression and neuronal hyperexcitability. Thus, understanding the interaction between brain tumor cells and neurons is critical to infer the mechanisms involved in tumor progression and neuronal hyperexcitability.

Study (ref.nr.)	Type of tumor/lesion	Animal	IUE‐embryonic day	System	Promoter	Construct
Low‐grade/benign						
Koh et al. [13]	GG	Mouse	E14	PB‐IUE	CAG	BRAF^V637E^
Goz et al. [141]	Hyperexcitable dysplastic cortical neurons	Mouse	E14.5	PB‐IUE	Nestin	BRAF^V600E^
Goz et al. [141]	Hyperexcitable dysplastic cortical neurons + astrogliosis (GN)	Mouse	E14.5	PB‐IUE	GLAST	BRAF^V600E^
Cases‐Cunillera et al. [125]	GG	Mouse	E14	PB‐IUE	CAG	BRAF^V600E^ + pAkt
Cases‐Cunillera et al. [125]	PLNTY	Mouse	E14	PB‐IUE	CAG	BRAF^V600E^
High‐grade/malignant						
Cases‐Cunillera et al. [125]	Anaplastic GNT	Mouse	E14	PB‐IUE	CAG	BRAF^V600E^ + pAkt + Trp53^KO^
Goz et al. [141]	GBM	Rat	E13.5	PB‐IUE	CAG	HRasV12/AKT
Chen et al. [142]	GBM	Rat	E13.5	PB‐IUE	GFAP	HRasV12/AKT
Chen et al. [142]	Oligoastrocytoma	Rat	E13.5	PB‐IUE	MBP	HRasV12/AKT
Zuckermann et al. [143]	GBM	Mouse	E13.5	PB‐IUE	–	gTrp53, gPTEN, gNf1
Patel et al. [144]	DIPG	Mouse	E13.5	PB‐IUE	CAG	PDGFB + DNp53 + H3.3K27M
Patel et al. [144]	DIPG	Mouse	E13.5	PB‐IUE	CAG	PDGFRA^D842V^ + DNp53 + H3.3 K27M
Patel et al. [144]	DIPG	Mouse	E13.5	PB‐IUE	CAG	PDGFRA^WT^ + DNp53 + H3.3 K27M
Pathania et al. [145]	HGG	Mouse	E12.5 (hindbrain) E13.5 (forebrain)	PB‐IUE	CAG	H3.3 K27M and Trp53 loss

#### 3.2.3 Limitations of IUE‐based models

Nevertheless, it is important to notice that IUE offers some limitations for developing these tumor models *in vivo*. First, IUE tumor models do not harbor tumor tissue from patients, which may be less convenient for personalized medicine for which PDX models represent an adequate technical platform [126]. Moreover, the optimal embryonic developmental timepoint for the IUE that ultimately will decide the targeted precursor cell population is arbitrary. Besides these limitations, the above‐mentioned aspects support that IUE animal models for LGNT can be of significant value for providing foundational insights into the crosstalk between tumor cells and neighboring neurons, which seems highly required for finding new antitumor and anticonvulsant therapies.

### 3.3 Molecular mechanisms sustaining the relationship formed between tumor and surrounding neurons in LGNT: A bidirectional process?

As mentioned above, several studies have provided evidence of interactions between LGNT cells and neurons. Mechanisms involved in epileptogenicity related to these tumors are starting to be elucidated based on recent progress in the identification of factors released by the tumor cells. A paracrine‐mediated effect appears to be very realistic in these tumors taking into account that they are very often associated with adjacent abnormal/epileptic cortical structures [57, 67, 101].

As reviewed in depth elsewhere, accumulating evidence suggests that immune and inflammatory factors, such as cytokines, may play an important role in epileptogenesis (reviewed in [127]). High levels of reactive microglia, which is the main source of cytokines in the brain, have been detected in human LGNTs and peritumoral regions and its activation correlates with epilepsy duration and seizure frequency [92, 128]. Other studies have also shown a strong expression of genes related to immune response and inflammation has been found in human brain specimens [121, 129, 130] as well as in mouse models for GNTs [125]. Especially, high expression of the proinflammatory cytokine interleukin 1β (IL‐1β) has been detected in human GNT specimens [120]. Given the association between IL‐1β and ictogenesis [131, 132, 133] this proinflammatory cytokine appears as a putative main factor to mediate epileptogenesis in these lesions. Interestingly, electrophysiological recordings showed that IL‐1β can enhance GABA‐ergic inhibition in human GG tissue [134].

High levels of extracellular glutamate released by gliomas are likely to be linked to neuronal excitability and impairment of the GABA‐ergic signaling contributes to seizure activity in benign and malignant gliomas [14, 15, 47, 135]. However, this aspect has not been experimentally evaluated in benign GNTs yet and only data on the changes in expression of glutamate and GABA receptors have been provided. A high expression of glutamate receptors was found in human GGs [63, 66]. Likewise, a reduction of GABAA receptors in the neuronal component has been found in human GGs [68, 136] as well as in the GG mouse model [137]. Moreover, KCC1 has been found upregulated in human GGs [46]. These results suggest that extracellular levels of glutamate and GABA neurotransmitters may be impaired and contribute to hyperexcitability in these tumors. Finally, growth factors, such as the brain‐derived neurotrophic factor (BDNF), which regulates neuronal network function [138, 139], and its receptor TrkB, have also been detected in high levels in human GG samples [140].

All these features likely contribute to seizure activity sustaining that many different mechanisms may be prone to cause ictogenesis. In contrast, while there is evidence of existing signaling from the tumor to the neurons, not much is known about the effect of the neurons on the tumors in LGNTs. Future studies will need to assess this aspect in depth to find therapies to target both the tumor and epilepsy‐associated mechanisms.

## 4 Conclusions and perspectives

Extensive efforts by neuropathologists and related scientists are continuously made to set the criteria for the classification of LGNTs, which is essential for the subsequent generation of animal models. Studies performed on PDX‐based tumor models built sufficient evidence to prove a dynamic interaction between tumor and neuronal cells. However, these studies required careful consideration since PDX‐based tumor models lack intrinsic connections with the brain parenchyma, which represents an important challenge for the study of mechanisms underlying the crosstalk between tumor cells and the neuronal compartment. In this regard, IUE becomes an essential tool for the development of brain tumor models recapitulating the crucial features of their human counterparts. Overall, revealing the mechanisms underlying the crosstalk between tumor and neurons may provide the basis for finding new molecules which are targetable to ultimately reduce seizure activity and suppress tumorigenesis.

## Conflict of interest

The authors declare no conflict of interest.

## Author contributions

SCC, AJB, and GHG conceived and designed the project; SCC, LLF, PM, AJB, and GHG drafted, edited and reviewed the manuscript.
